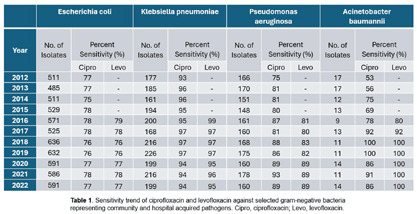# Prolonged Decrease in Fluoroquinolone Utilization and Associated Reversal to High Level Sensitivity of P. aeruginosa and A. baumannii

**DOI:** 10.1017/ash.2025.249

**Published:** 2025-09-24

**Authors:** Andrew Dysangco, Tamra Pierce, Dawn Bravata

**Affiliations:** 1 Joseph Maxwell Cleland Atlanta Veterans Affairs Medical Center; 2 Update

## Abstract

**Background:** Our antibiotic stewardship program (ASP) has decreased inpatient fluoroquinolone (FQ) consumption using a prior-authorization process and physician-led audit and feedback. The objective of this project was to examine if the decrement in FQ use was associated with improvement in the FQ sensitivity rate of selected gram-negative organisms. **Method:** Quarterly days of therapy standardized to 1000 care-days (DOT/1000 CD) for ciprofloxacin, moxifloxacin, and levofloxacin were calculated as the measure of inpatient FQ consumption starting from quarter three of fiscal year 2012 to the end of fiscal year 2022. To evaluate resistance patterns, facility-level sensitivity rates for ciprofloxacin and levofloxacin against Escherichia coli, Klebsiella pneumoniae, Pseudomonas aeruginosa, and Acinetobacter baumannii were collected from the antibiogram published from calendar year 2012-2022. FQ sensitivities for the first half of the study period (2012-2016) were compared to the second half (2017-2022) using Chi-square tests. **Results:** Inpatient FQ consumption from 2012-2022 is summarized in Figure 1. After the initiation of the ASP in 2012, a gradual and steady decline in in-patient FQ use was observed. During the first four quarters, the average FQ use was 71.3 DOT/1000 CD. Lowest use was observed during 2020 calendar year, with average consumption of 19 DOT/1000 CD. FQ consumption then increased to 30.6 DOT/1000 CD (Q2-2021 to Q4-2022). Overall, there was a decrease of 40.7 DOT/1000 CD; a 56.16% decrease in FQ consumption over the 11-year period. E. coli and K. pneumoniae sensitivity to FQs remained stable over time with a mean of 77% and 95%, respectively (see Table 1). We observed statistically significant improvements in FQ sensitivity for P. aeruginosa from 75% to 89% (p-value **Conclusions:** We found differing effects of decreased inpatient FQ use on antibiogram sensitivities between four gram-negative organisms. Significant improvements in FQ sensitivity were observed in P. aeruginosa and A. Baumannii reverting to high level sensitivity but not for E. coli or K. pneumoniae. These data support the effectiveness of ASPs in reducing FQ use and improving some FQ sensitivities.

**Figure:**
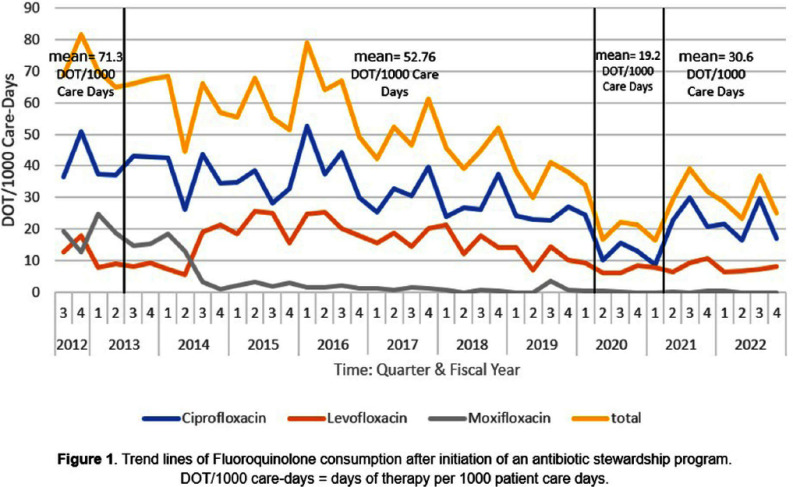

**Figure:**